# Enhanced Denitrification of Integrated Sewage Treatment System by Supplementing Denitrifying Carbon Source

**DOI:** 10.3390/ijerph18189569

**Published:** 2021-09-10

**Authors:** Dongkai Chen, Peizhen Chen, Xiangqun Zheng, Weimin Cheng, Qiang Wang, Xiaocheng Wei

**Affiliations:** Agro-Environmental Protection Institute, Ministry of Agriculture and Rural Affairs, Tianjin 300191, China; chendongkai2020@126.com (D.C.); Zhengxiangqun@126.com (X.Z.); wmcheng@126.com (W.C.); wq319320@126.com (Q.W.); weixiaocheng@aepi.org.cn (X.W.)

**Keywords:** integrated sewage treatment system (ISTY), supplementary carbon source, carbon release mechanism, denitrification, nitrogen removal

## Abstract

Integrated sewage treatment system (ISTY) is a new technology for rural domestic sewage treatment. In the ISTY, the carbon source in the denitrification stage is often insufficient, affecting the denitrification efficiency. In order to improve the denitrification efficiency, several commonly available agricultural wastes, peanut shell (PS), sawdust (SD), peat (PT), and their mixtures (MT), were selected as supplementary carbon sources in the denitrification stage of ISTY to study the denitrification efficiency. Results show that PS exhibited a high carbon release capacity. PS released an enormous amount of carbon in 144 h, and the cumulative total organic carbon was 41.99 ± 0.7 mg/(g·L). The optimum carbon source dosage was 3 g/L, the nitrate removal rates of PS exceeded 95% after 48 h, and the denitrification rates were 9.35 mg/(g·L), which were 63.92% higher than that of the control group. After running the ISTY for 120 h, and with PS as supplementary carbon sources, the removal rate of TN increased from 29.76% to 83.86%. At the genus level, the dominant denitrifying bacteria in ISTY, after adding PS, were *Pseudomonas* and *Cupriavidus*, accounting for 78.68%, an increase of 72.90% compared with the control group. This evidence suggested that PS can obviously enhance the denitrification efficiency of the ISTY as a supplementary carbon source.

## 1. Introduction

Integrated sewage treatment system (ISTY) is a new technology for rural domestic sewage treatment. The ISTY integrates the independent nitrification-denitrification process in the same reactor to achieve integration, miniaturization, and operation convenience. The ISTY used in this experiment was composed of filter, adsorption, aerobic, denitrification, and filter tanks, in a series, and adopted micro-power operation. Gravel filled the filter tank, and vermiculite filled the adsorption tank. However, the rural sewage, with a low C/N ratio (3:1–5:1), cannot satisfy the carbon source demand of denitrifying microorganisms in the ISTY, thus affecting the nitrogen removal efficiency of the ISTY. Given insufficient influent carbon source, high-quality carbon source, such as glucose, lactose, methanol, and acetic acid, is added to improve the sewage C/N [1,2]. However, adding high-quality carbon sources has some disadvantages, such as high cost and inconvenient transportation [3,4,5], and produces excessive sludge in the treatment system. Consequently, this method has some disadvantages in practical application [6].

Using agricultural waste as a solid carbon source is a relatively new method of denitrification enhancement [7,8]. Compared with high-quality carbon source, agricultural waste can not only be used as an additional carbon source but also provide a carrier for the growth of, and a stable living environment for, microorganisms [9,10]. Ling et al. [11] studied the carbon release and nitrogen removal performance of six kinds of agricultural wastes, namely, rice straw, wheat straw, corn straw, corncob, soybean stalk, and soybean hull, as carbon sources. They found that corncob has a strong carbon release and denitrification performance and does not easily lead to secondary pollution risk. Liang et al. [8] found that using rice straw as an external carbon source has a good effect on the nitrogen removal of agricultural wastewater with a low nitrate load. At present, the research on the addition of carbon sources mainly focuses on the experimental simulation and ecological treatment of agricultural wastewater. However, agricultural waste as a supplementary carbon source, in the ISTY of rural domestic sewage, has received little attention.

Peanut shell (PS), sawdust (SD), peat (PT), and their mixtures (MT) have no biological toxicity and a large specific surface area, which can be used not only as an external carbon source, but as a carrier of biological attachment. In addition, in rural areas, PS, SD, and PT have a wide range of sources and low cost, so using them as external carbon sources can not only improve the efficiency of nitrogen removal, but it can also effectively resource the waste. Therefore, PS, SD, PT, and MT were selected as additional carbon source materials to study their carbon release mechanism and nitrogen removal efficiency. On this basis, four kinds of carbon sources were studied as supplementary carbon sources, in the denitrification stage of the ISTY, to strengthen the performance and economic benefits of integrated sewage treatment equipment.

## 2. Materials and Methods

### 2.1. Materials

The surface impurities of PS, SD, PT, and MT were washed with deionized water. Then the materials were dried in the oven at 55 °C. The dried materials were crushed and ground to 3–5 mm and placed in sealed bags. MT was prepared by evenly mixing PS, SD, and PT at 1:1:1.

### 2.2. Methods

#### 2.2.1. Release Experiments

The four kinds of carbon sources were placed (3 g) in separate 2000-mL conical bottles to which 1000 mL of deionized water was added. The bottle mouth was sealed with a rubber stopper. After 3, 12, 24, 48, 72, 96, 120, and 144 h of the experiment, samples were taken. The water changed thoroughly after each sampling (performed in triplicates). The extracted solutions were used to determine the chemical oxygen demand (COD), the total organic carbon (TOC), the total nitrogen (TN), and the total phosphorus (TP).

#### 2.2.2. Denitrification Performance of Carbon Source

##### Selection of Optimal Carbon Source Dosage

The four carbon sources were weighed and added to the test water distribution at 1, 3, 6, and 9-g/L dosages. The water used in the test was configured with KNO_3_, KH_2_PO_4_, MgSO_4_, NaCl, and a certain amount of trace elements. The water composition is shown in Table 1. Liquid samples were taken after 3, 12, 24, 48, 72, 96, and 120 h to determine the COD and TN contents.

##### Determination of Denitrification Performance

In separate 2000-mL conical bottles, 3 g each of the carbon sources were added, followed by 0.1 L of activated sludge and 1.0 L of test sewage. Four carbon source addition treatments (i.e., PS, SD, PT, and MT) were set up, and the control treatment had no additional carbon treatment. The experiments were performed in triplicates. The NO_3_^−^-N, TN, and COD contents in the conical bottles were determined at 6, 12, 24, 48, 72, 96, and 120 h. Quality and formula of distributed water was followed by Table 1. The activated sludge used was collected from the anoxic tank of a sewage treatment plant in Tianjin, China.

#### 2.2.3. Enhanced Denitrification Effect of ISTY by Supplementing Carbon Sources

**Figure 1:**
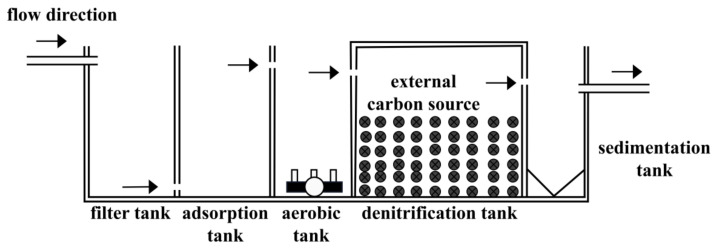
Reactor diagram of the integrated sewage treatment system.

The batch experiment on actual rural domestic sewage treatment was conducted using the self-designed ISTY. The test device is shown in Figure 1. The reactor is made of plexiglass with the following features: length × width × height = 90 cm × 30 cm × 45 cm, and the effective volume is 115.0 L. From left to right, the system has a filter tank, an adsorption tank, an aerobic tank, a denitrification tank, and a sedimentation tank, with gravel to fill the filter tank and vermiculite to fill the adsorption tank. The sewage passed through the filter, adsorption, aerobic, denitrification, and sedimentation tanks. The carbon source was added to the denitrification tank. After 6, 12, 24, 48, 72, 96, and 120 h of operation, samples of the influent and effluent of the denitrification tank were taken to determine the TN, NH_4_^+^-N, and NO_3_^−^-N contents and the microbial diversity. This experiment used sewage taken from the rural sewage in a suburb of Tianjin, China. The influent quality index of the denitrification tank is shown in Table 2.

#### 2.2.4. Economic Benefit Evaluation of Adding Carbon Source in Denitrification Stage of ISTY

Using agricultural waste as the supplementary carbon source to strengthen denitrification and reusing reclaimed water after treatment can effectively save water resources, reduce sewage discharge, improve the environment, improve the utilization rate of water resources, and bring economic benefits. Reclaimed water is used instead of tap water in farmland irrigation and greening, with common and cheap agricultural waste replacing soluble low molecular weight organic compounds to enhance the denitrification stage, resulting in economic savings.

The following formula can calculate the economic benefits:

*C = C_1_ + C_2_,* (1)

where *C* represents the total economic benefit ($), *C*_1_ represents the financial benefit brought by water savings ($), and *C*_2_ represents the economic benefit obtained from energy savings ($).

The evaluation formula of economic benefit from water savings is as follows:

*C_1_ = (A_1_ − A_2_)Q_1_,* (2)

where *A*_1_ is the price of tap water ($/m^3^), *A*_2_ is the price of reclaimed water ($/m^3^), and *Q*_1_ is the amount of sewage treatment (m^3^).

The evaluation formula of economic benefit from energy savings is as follows:

*C_2_ = (D_1_ − D_2_)Q_2_,* (3)

where *D*_1_ is the cost of soluble low molecular organic matter to be added per unit of sewage ($), *D*_2_ is the cost of common agricultural waste to be added per unit of sewage ($), and *Q*_2_ is the amount of sewage treatment (m^3^).

### 2.3. Characterization and Analytical Methods

The C, N, P, and S contents were determined using an elemental analyzer. The TOC analyzer was used to measure the total organic carbon (TOC). The COD, NO_3_^−^-N, NO_2_^−^-N, NH_4_^+^-N, TN, and TP contents were analyzed according to relevant standard methods [12,13]. The changes of functional group absorption peaks before and after carbon release from agricultural waste were reflected by infrared spectroscopy (FTIR). The agricultural wastes were determined by Fourier transform near infrared spectrometer, each sample was scanned by near-infrared spectroscopy for 64 times and repeated for 3 times. The average of the three measurement results was taken as the final spectral value of the sample. The surface property of the agricultural wastes was observed through scanning electron microscopy (SEM), infrared spectroscopy, and X-ray energy spectroscopy (EDS). The microbial diversity was detected by high-throughput sequencing. The extracted DNA was amplified by PCR using 16S rRNA fine bacteria common primers 5′-ACTCCTACGGGAGGCAGCA-3′ and 5′-GGACTACHVGGGTWTCTAAT-3′ (v3-v4 region), and all the sequence readings were clustered into the operational classification unit (similarity threshold is 97%) for subsequent species annotation and functional analysis.

### 2.4. Data Analysis Method

The experimental data were sorted in Microsoft Excel 2019 (Microsoft, Redmond, WA, USA) and analyzed by SPSS 25.0 (IBM corporation, Armonk, NY, USA) with significant differences (*p* < 0.05). The Origin 2019 software was used for drawing.

## 3. Results and Discussion

### 3.1. Release Experiments

#### 3.1.1. Carbon Source Element Content Analysis

The C, N, and P in agricultural wastes demonstrate not only the release capacity of carbon sources but also the potential risk of secondary pollution. The higher the C content is, the greater the potential of carbon release is. As shown in Table 3, the C content in the three materials is between 44.18% and 50.04%, indicating that each material has a specific feasibility as a carbon source to promote the denitrification process. The N, P, and S contents can reflect the potential risk of secondary pollution release. The N, P, and S contents in PT were the highest (i.e., 2.27%, 0.34%, and 0.39%, respectively), indicating that PT, as an external carbon source, has a specific risk of releasing pollutants, such as N, P, and S, thus affecting the water quality of the effluent. The N, P, and S contents in PS (i.e., 1.20%, 0.18%, and 0.30%, respectively) were lower than those in PT, and the potential risk of secondary pollution caused by PS is relatively low.

#### 3.1.2. Carbon Release Performance of Carbon Source

**Figure 2:**
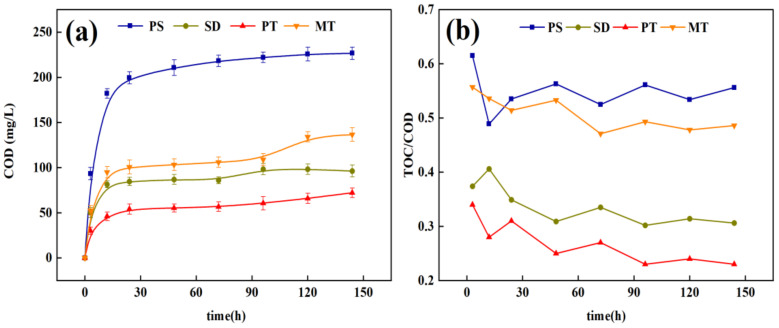
Carbon release curves and TOC/COD of different carbon sources: (**a**) Carbon release curves of different carbon sources; (**b**) TOC/COD of different carbon sources. Note: TOC: total organic carbon; COD: chemical oxygen demand.

The cumulative carbon release characteristics (measured by COD) of the different carbon sources are shown in Figure 2a. The materials have similar carbon release properties but released different amounts of carbon sources. In the initial 6 h, the cumulative amount of the carbon sources increased quickly, and then, the release velocities stabilized at very low levels in 6–144 h. This phenomenon occurred because the surface of agricultural waste contains substantial soluble organic carbon [14] and organic particles that easily fall off [15], and these substances easily dissolve in water and release organic carbon. As the reaction proceeds, the cellulose, which is difficult to degrade in agricultural waste, begins to decompose, leading to slow carbon release until equilibrium. Ling et al. [11] studied the carbon release performance of six different agricultural wastes. Their results show that the six agricultural wastes’ carbon release process can be divided into the rapid and slow-release stages, which are consistent with the results of this study. In terms of carbon release amount, PS, SD, PT, and MT displayed specific differences. The order of total carbon release amounts in 144 h is as follows: PS > MT > SD > PT. The accumulated carbon release amounts were 226.6 ± 6.9 mg/L, 136.6 ± 7.5 mg/L, 96.2 ± 6.6 mg/L, and 72.2 ± 5.2 mg/L, respectively. The carbon release amount and rate of PS are the highest among the four materials.

TOC can reflect the number of carbon sources available to microorganisms better than COD. The TOC/COD value can directly reflect the proportion of carbon sources that can be used by microorganisms. The average TOC/COD ratios, in the leaching solution of the four carbon sources, is as follows: PS (0.55) > MT (0.51) > SD (0.34) > PT (0.27). In the study by Xiong et al. [16], the average TOC/COD ratio of PS extract within 180 h was 0.59, which is consistent with the average TOC/COD of PS in this study. Figure 2b shows that the TOC/COD of PS is larger than that of SD, PT, and MT, indicating that the organic carbon released by PS is relatively higher, and more organic carbon can be used in the process of denitrification. On the contrary, the relatively low organic carbon released by PT may lead to the risk of high COD in the effluent.

To further understand the specific mechanism of carbon release by different materials, the carbon release process of four different materials was fitted by the first-order kinetic and Ritger–Peppas equations.

The first-order kinetic equation is as follows:

(4) $$ln\frac{M_{\infty }-M_{t}}{M_{\infty }}=-kt,$$

where *M_t_* is the carbon release amount at time t (mg/kg), *M_∞_* is the maximum carbon release amount (mg/kg), and *K* is the rate constant of carbon release.

The Ritger–Peppas equation is as follows:

(5) $$\frac{M_{t}}{M_{\infty }}=kt^{N},$$

where *M_t_* is the cumulative release amount at time t (mg/kg), *M_∞_* is the total release amount (mg/kg), *K* is the carbon release rate constant, and *N* is the carbon release index, which represent the carbon release mechanism. When the value of *N* is less than 0.45, the diffusion process is dominant. When the value of *N* is between 0.45 and 0.89, the main mechanisms are diffusion and material skeleton dissolution. When the value of *N* is greater than 0.89, the mechanism is mainly a dissolution process [17].

The carbon release processes of the four carbon sources satisfy the first-order kinetic and Ritger–Peppas equations (show in Table 4). The fitting results of each carbon source have a high correlation. According to the first-order kinetic equation fitting results, the surface molecules of the carbon sources in contact with water were hydrolyzed into micromolecules, and the rate of hydrolysis is proportional to the surface area [16]. The *K* values of the four carbon sources show no obvious gap, indicating that four carbon sources have the same carbon release rate. According to the fitting results of the Ritger–Peppas equation, the *N* values of the carbon sources are less than 0.45, indicating that the carbon release matrix is mainly in the diffusion process.

#### 3.1.3. Release of Nitrogen and Phosphorus from Carbon Sources

**Figure 3:**
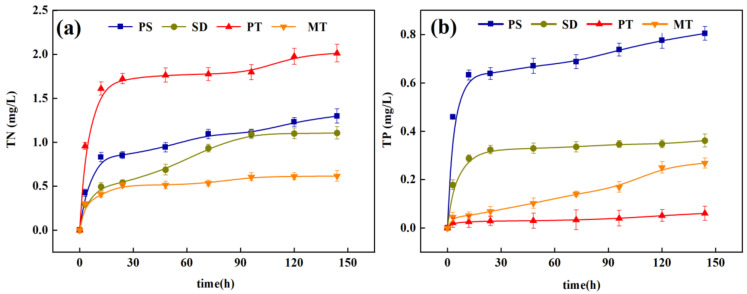
Release of total nitrogen (TN) and total phosphorus (TP) of different carbon sources; (**a**) TN; (**b**) TP.

The process of releasing carbon from plant hydrolysis also releases certain nitrogen and phosphorus elements into the water, and the risk of secondary release of pollutants exists [18]. Figure 3 presents the cumulative release of TN and TP in the leaching solutions of the four carbon sources. The cumulative release rule of TN and TP, in the leaches of the four carbon sources, is the same as that in Section 3.1.2, with the rapid release period from 1 h to 6 h and the slow-release period after 6 h. All four carbon sources release N and P to some extent. However, the total release amount is small, slightly affecting the denitrification stage and the entire wastewater treatment process. This phenomenon occurs because the N and P groups in each carbon source are not easy to hydrolyze. In addition, it has certain adsorption properties, resulting in minimal nitrogen and phosphorus in the leaching solution. The four carbon sources all have definite carbon release performance, and the TN and TP release amount is small. The risk of secondary pollution is less, in enhanced denitrification, as an external carbon source.

#### 3.1.4. FT IR Characteristics of Carbon Source before and after Carbon Release

**Figure 4:**
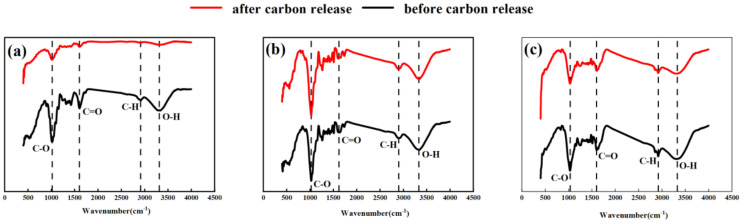
FTIR spectra before and after carbon release of different carbon sources. (**a**) peanut shell (PS); (**b**) sawdust (SD); (**c**) peat (PT).

The infrared spectroscopy (FT IR) spectra of PS, SD, and PT before and after carbon release at a wavenumber of 500–4000 cm^−1^ are shown in Figure 4. Wavenumbers 3333.36, 3327.33, and 3320.10 cm^−1^ show the vibration peaks formed by the O-H stretching vibration [19]. The peaks at wavenumbers 2918.74, 2916.32, and 2905.72 cm^−1^ belong to the C-H symmetric stretching vibration absorption and the in-plane bending vibration [20]. The absorption peaks at wavenumbers 1625.94, 1603.28, and 1600.87 cm^−1^ belong to the C=O stretching vibration. The absorption peaks, at wavenumbers 1243.86, 1031.48, and 1026.43 cm^−1^, are related to C-O [21]. The comparison of the functional group distributions of PS, SD, and PT before carbon release indicates a similarity. After carbon release, the absorption peaks of SD and PT were insignificant, indicating that their chemical bonds were stable. The absorption peak intensities of the O-H, C-H, C=O, and C-O of PS changed considerably (Figure 4a), indicating that many carbon-containing chemical bonds were broken after PS immersion, which can release definite organic carbon and provide a carbon source for denitrification.

### 3.2. Denitrification Performance of Carbon Source

#### 3.2.1. Screening of Carbon Source Dosage

**Figure 5:**
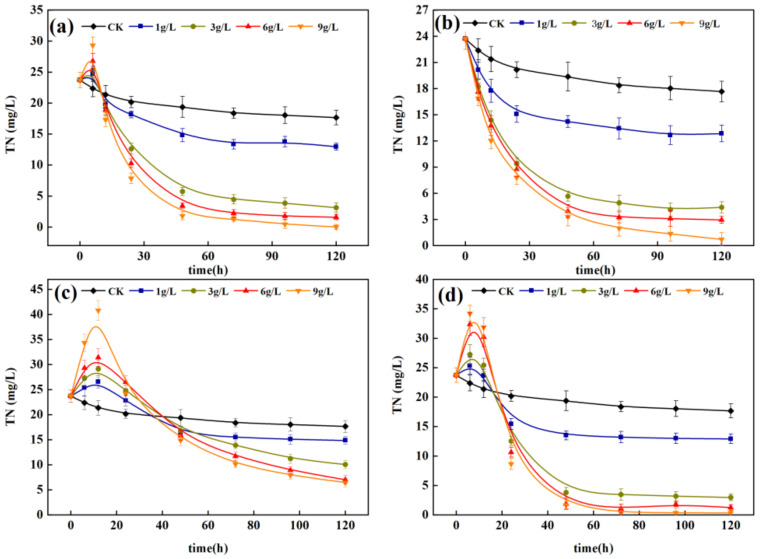
TN removal effect in water body under different carbon source dosage. (**a**) PS; (**b**) SD; (**c**) PT; (**d**) MT.

**Figure 6:**
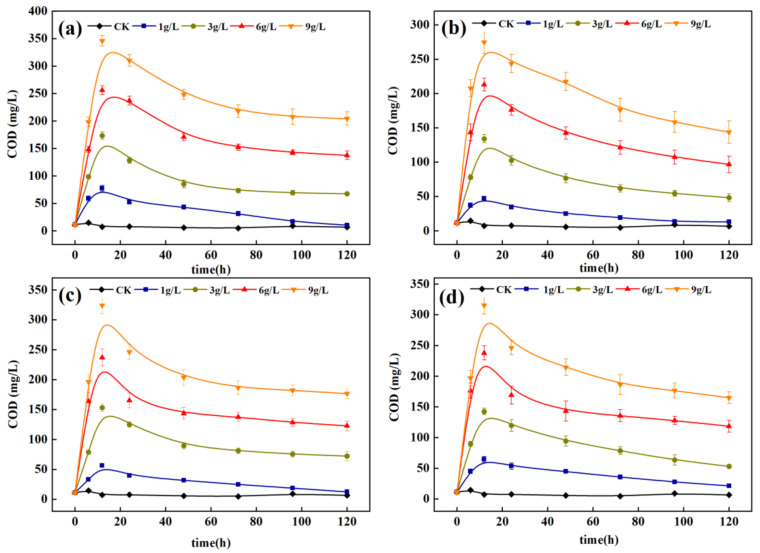
Changes of COD in water under different carbon source dosages. (**a**) PS; (**b**) SD; (**c**) PT; (**d**) MT.

The TN removal effect in water, by each carbon source at different dosage levels, is shown in Figure 5. When the dosage was 1 g/L, the TN concentration of each carbon source decreased continuously in 0–24 h because the water had sufficient carbon sources for nitrification and denitrification. After 24 h, the carbon source content in the water was insufficient, and the denitrification capacity decreased, decelerating, and then maintaining the TN removal rate. When the dosage was 3, 6, and 9 g/L, the TN content in water decreased, obviously, from 0 h to 48 h. From 48 h to 120 h, the TN removal rate gradually decreased. When the PS, SD, and MT dosages exceeded 3 g/L, the removal rate of TN was high. Dosages 3, 6, and 9 g/L exhibited little difference in TN removal rate in water. However, dosages 6 and 9 g/L significantly (*p* < 0.05) increased the COD concentration in the effluent (Figure 6), resulting in secondary pollution risk. Thus, the optimal dosage of carbon source is 3 g/L.

#### 3.2.2. Denitrification Performance

**Figure 7:**
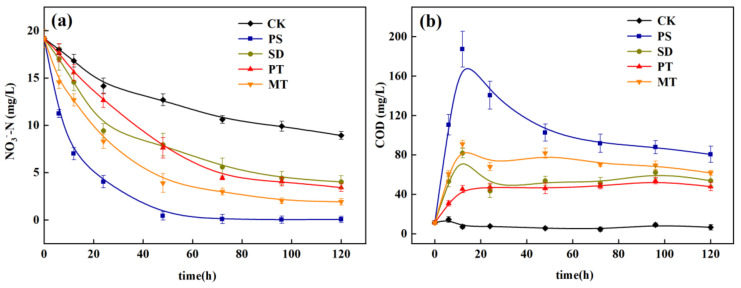
Changes of NO_3_^−^-N and COD in denitrification process; (**a**) NO_3_^−^-N; (**b**) COD.

The variation trends of NO_3_^-^-N and COD treated by the four carbon sources are shown in Figure 7. Compared with CK, the addition of four carbon sources enhanced the denitrification effect. In 0–48 h, the concentration of NO_3_^−^-N decreased rapidly, and the removal rate increased. From 48 h to 120 h, the concentration of NO_3_^−^-N decreased gently, and the removal rate decreased gradually. This phenomenon occurred because each carbon source released enough organic carbon 0–48 h for denitrifying microorganisms to reduce NO_3_^−^-N to N_2_ or N_2_O, which was proven in Experiment 2.3.2. With the gradual utilization of organic carbon, the removal rate of NO_3_^−^-N by denitrifying microorganisms also decreased gradually. The removal process of NO_3_^−^-N by the four carbon sources is correlated with the release process of COD from each carbon source in Section 3.1.2, indicating that the release of COD played an important role in the removal of nitrate in the water. During denitrification, the change trend of COD increased rapidly from 0 to 12 h, decreased gradually from 12 h to 48 h, and gradually stabilized after 48 h. This result can further explain the variation trend of NO_3_^−^-N removal during denitrification. Carrera [22] found, through a wastewater denitrification test, that adding a carbon source enables the C/N in water to exceed 7 and reach efficient denitrification. In this test, the average COD/N ratios of the carbon sources in water follow the order PS (10.4) > MT (7.3) > SD (4.9) > PT (3.3) > CK (0.5). The average C/N of PS and MT can exceed 7. The NO_3_^−^-N removal rate of PS can reach 97% at 48 h, and that of MT can reach 90% at 120 h, which are both higher than that of the control group. Xiong et al. [16] studied the denitrification performance by adding various agricultural wastes and synthetic macromolecules, and they found that adding PS to nitrate water could achieve near-complete denitrification within 48 h, which is similar to the experimental results in this study.

The COD in each experimental treatment group increased, with time, to different degrees, then decreased gradually and tended to be gentle. The carbon sources had significant (*p* < 0.05) effects on the COD in water. Among them, the carbon supply of PS was sufficient, increasing to 187.5 mg/L in 12 h, gradually decreasing to 82.3 mg/L in 12–72 h, and then stabilizing between 80 and 90 mg/L. After 48 h, the COD in the water tended to be stable, and the removal rate of nitrate also gradually tended to be stable. This phenomenon indicates that each carbon source released TOC within the initial 48 h, improving the ability to denitrify microorganisms. For the PS, the nitrate in water decreased from 20 mg/L to 0.4 mg/L in 48 h. Compared with SD and PT, the nitrate content only decreased to approximately 8 mg/L. In addition, the nitrite content in water, treated by each carbon source, was nearly less than 1 mg/L (data not shown in this paper), indicating that the organic substances released by carbon sources material can be easily used as denitrifying microorganisms [18].

#### 3.2.3. Characteristics of Surface Structure Change of Carbon Source

**Figure 8:**
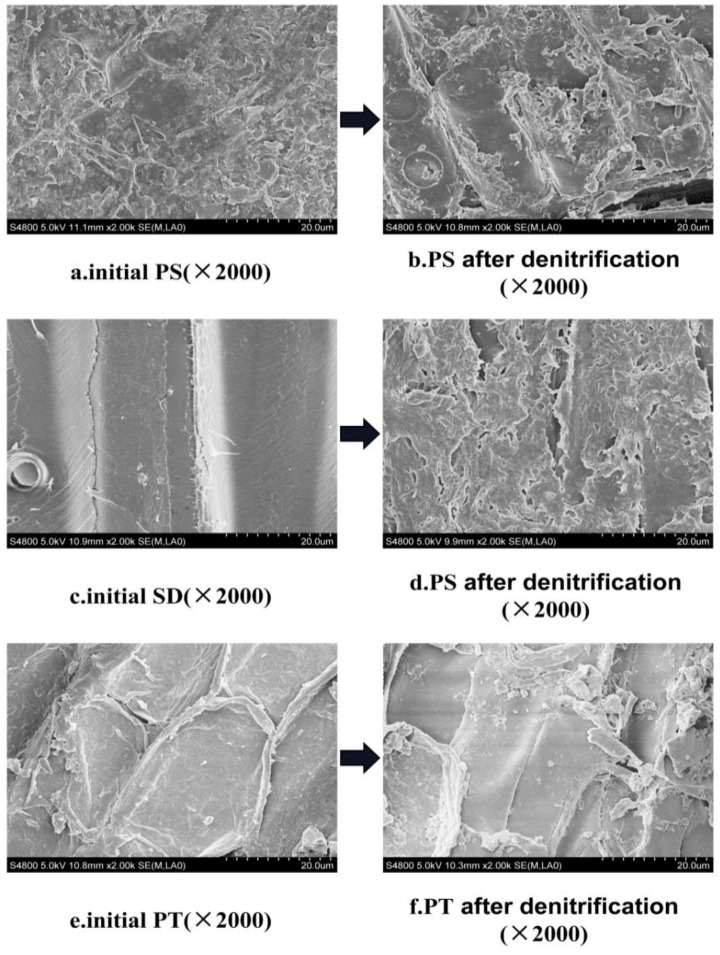
Scanning electron microscope (SEM) images of each carbon source before and after denitrification.

**Figure 9:**
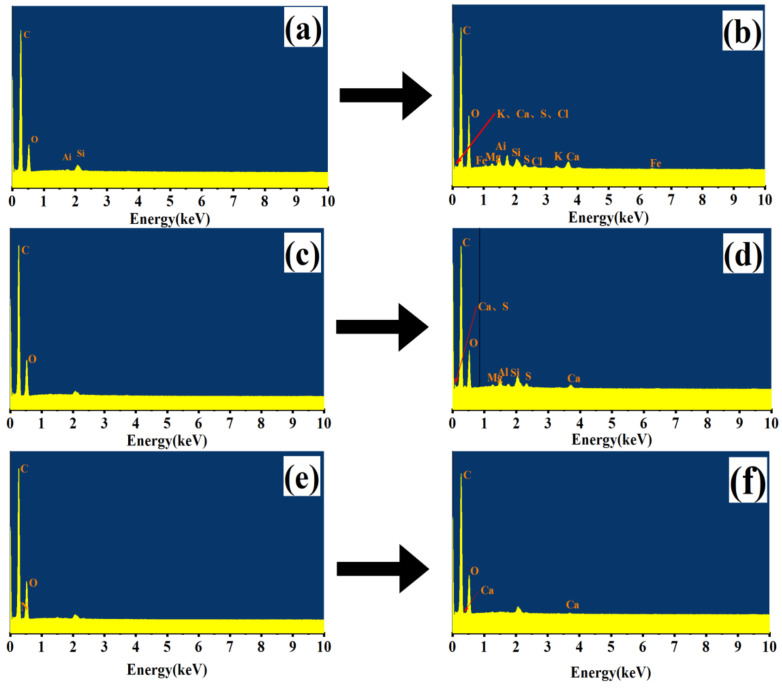
Energy dispersive spectrometer (EDS) images of each carbon source before and after denitrification. (**a**) initial PS; (**b**) PS after denitrification; (**c**) initial SD; (**d**) SD after denitrification; (**e**) initial PT; (**f**) PT after denitrification.

The morphological structure of materials has an important effect on the adhesion of microorganisms. Materials with certain surface roughness can provide favorable conditions for the adhesion of microorganisms. The rougher the material surface is, the larger the specific surface area is, and thus, it is more conducive to the attachment of microorganisms for further improvement of the denitrification performance [23]. The results obtained by SEM and EDS are shown in Figure 8 and Figure 9. As can be seen from Figure 8, the surface roughness of the initial carbon source materials follows the descending order PS > PT > SD. The surface of PS is the roughest and has many protrusions, which are conducive to the attachment of microorganisms. The surface of PT has some protrusions. The surface of SD is the smoothest, which is not conducive to the attachment and growth of microorganisms. After 120 h of denitrification, the roughness of the surface of the three materials increased after hydrolysis and biodegradation. The surface structure of PS was damaged to a certain extent, and many voids were produced, indicating that the hydrolysis degree of PS was distinct. The utilization degree of PS was high, and the further increase in specific surface area is suitable for the long-term membrane growth of microorganisms to improve the denitrification performance. After denitrification, the surface structure of SD became rough, and many microorganisms attached to it. However, the roughness of the PT surface changed slightly after denitrification, and the amount of microbial attachment on the PT surface decreased, indicating that the degree of microbial attachment and utilization of PT was low.

Figure 9 shows that only a few elements are on the surface of the three different carbon sources at the beginning. C, O, Al, and Si can be detected on the surface of PS, while only C and O can be found on the surface of SD and PT. After denitrification, different elements were added to the surface of the three materials. The PS surface showed the most elements, including Na, Ca, Fe, S, K, Mg, and Cl. The surface of SD showed Mg, Al, Si, Ca, and S elements. Meanwhile, only the Ca element was added to the PT surface. The elements added after denitrification are necessary trace elements for the growth and reproduction of microorganisms, indicating that each carbon source can provide organic carbon and a carrier for the attachment and growth of microorganisms. According to the types and quantities of trace elements attached to the surface of each material after denitrification, PS showed seven elements, SD showed five elements, and PT showed only one element. The results show that PS is conducive to the growth of microorganisms and thus enhances denitrification. PT is not conducive to the attachment, growth, and reproduction of microorganisms, which is also consistent with the previous SEM results.

### 3.3. Enhanced Denitrification Effect of ISTY by Supplementing Carbon Sources

#### 3.3.1. Effect of Carbon Source Supplement on Denitrification Enhancement of ISTY

**Figure 10:**
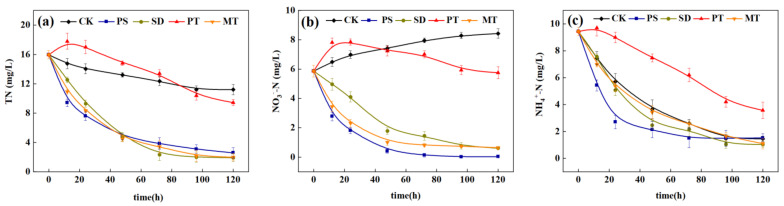
Effects of different carbon sources on denitrification performance in integrated treatment system. (**a**) total nitrogen concentration; (**b**) nitrate concentration; (**c**) ammonium nitrogen concentration.

When the dosage was 3 g/L, the denitrification effect of different carbon sources on the actual wastewater treatment in the denitrification stage of the ISTY is shown in Figure 10. In the control group, NO_3_^−^-N showed a gradual increase from 0 to 120 h because the nitrifying bacteria in the water oxidized ammonium NH_4_^+^-N to NO_3_^−^-N. However, the C/N was low, and the denitrifying bacteria had insufficient carbon source to reduce NO_3_^−^-N, resulting in the gradual increase of NO_3_^−^-N in water. The addition of carbon sources can significantly (*p* < 0.05) improve the NO_3_^−^-N removal rate. The removal rate of NO_3_^−^-N in water increased from −26.6% to 93.1% after PS addition, and the removal rate of NO_3_^−^-N was higher than that of other treatment groups. After 120 h of ISTY operation, PS were used as supplementary carbon sources. Consequently, the TN removal rate increased from 29.76% to 87.43%, and the accumulation of nitrate nitrogen and nitrous nitrogen decreased (not shown in this paper). However, the removal of TN by PT exhibited no significant difference compared with that by CK, mainly because PT has different forms of nitrogen, which were released into the water to increase the TN content [24]. PS have good TN and NO_3_^−^-N removal efficiencies, and the nonlinear equation fitting, of their variation with time, is shown in Table 5. PS have an excellent fitting effect, and the removal rate of NO_3_^-^-N and TN shows a good regression relationship with time (R^2^ > 0.95). Regarding the influence of NH_4_^+^-N, PS can increase the removal efficiency of NH_4_^+^-N, and the removal rate of NH_4_^+^-N can be increased from 39.2% to 71.4% at 24 h. This phenomenon occurs because the surface of PS is rough and can produce a definite physical adsorption effect. In addition, the rough surface can also be used for nitrifying and denitrifying microorganisms to grow on to improve the NH_4_^+^-N removal efficiency.

#### 3.3.2. Effects of Carbon Source Supplementation on Denitrifying Microorganisms

**Figure 11:**
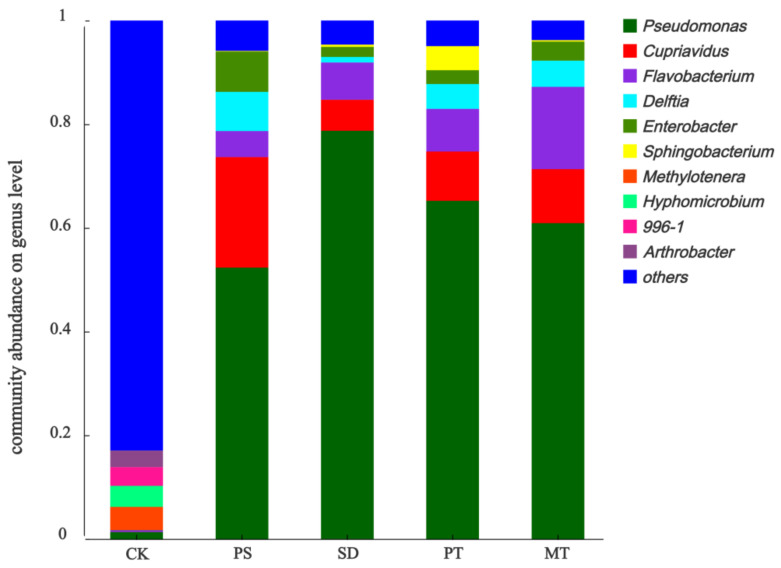
Community structure of bacteria, at genus level, after treatment with different carbon sources.

With the addition of a carbon source, the microbial community in the system changed observably in species composition and abundance compared with the control group (Figure 11). The top 10 bacteria with a systematic abundance of carbon source treatment include *Pseudomonas*, *Cupriavidus*, *Flavobacterium*, *Delftia*, *Enterobacter*, and *Sphingobacterium*, accounting for 94.16–96.24% of the total species. The main functions of these bacteria were related to nitrogen removal, and only 17.07% of the control group were related to nitrogen removal. The dominant bacteria in the carbon source addition group were *Pseudomonas*, *Cuprophylaxis*, and *Flavobacteria*. *Pseudomonas* has an aerobic denitrification ability, a good removal effect on NO_3_^−^-N, and a high removal efficiency [25]. *Cupriavidus* is capable of heterotrophic nitrification and aerobic denitrification. This bacteria has a good removal effect and a high removal efficiency for NH_4_^+^-N and NO_3_^−^-N, and it is capable of simultaneous nitrification and denitrification [26,27]. *Flavobacterium* is also a common denitrifying bacteria in wastewater treatment and has a high nitrate removal capacity [28]. Cydzik-kwiatkowska et al. [29] studied the effect of the C/N ratio in the indirect aeration granular sludge reactor on the nitrogen-converting communities induced in the supernatant of nitrification tank. Their results show that the C/N ratio determines the species composition of denitrifying bacteria. The abundance of *Pseudomonas* increases with the C/N ratio, and it has a high environmental tolerance. Meanwhile, the abundance of *Flavobaterium* increases with the C/N ratio. Hu et al. [30] studied the community structure of activated sludge in anaerobic wastewater treatment equipment and found that *Pseudomonas* plays a major role in wastewater nitrogen removal. Tang et al. [31] studied the application of *Pseudomonas* in constructed wetland for wastewater treatment and found that *Pseudomonas* could effectively degrade the nitrogen in wastewater. Furthermore, isolated *Pseudomonas* can decompose various chemicals and degrade difficult to degrade compounds under the condition of maintaining normal metabolism [32]. In addition to *Pseudomonas*, *Cupriavidus* also had high abundance in each carbon source treatment group, and the highest in PS treatment group. The results showed that the addition of carbon source promoted the biological activity of *Cupriavidus* and improved the denitrification efficiency. This conclusion has also been found in other studies. Jun et al. [33] studied the purification effect of *Cupriavidus* isolated from oligotrophic reservoir on polluted river water, indicating that the copper greedy bacteria has the ability of simultaneous nitrogen removal. Sun et al. [27] studied the performance of the isolated *Cupriavidus*. The results showed that the bacteria had heterotrophic nitrification and high removal efficiency for NH_4_^+^-N, NO_3_^—^N, and NO_2_^−^-N. Therefore, these results further indicate that adding a carbon source in the ISTY denitrification stage is conducive to the growth and reproduction of denitrifying bacteria, and it effectively strengthens the overall nitrogen removal effect.

#### 3.3.3. Economic Benefit Analysis of Adding Carbon Source in Denitrification Stage of ISTY

The lack of a carbon source has become an important factor restricting the efficiency of biological nitrogen removal. At present, several carbon sources are mainly used in wastewater treatment plants, including sodium acetate, methanol, and glucose. Although good results have been achieved, high cost remains a problem. In this study, agricultural waste was used in the system as an additional carbon source, resulting in cost savings from carbon sources and recycling waste. Section 3.3.1 indicates that the wastewater treated by ISTY can reach the quality standard of reclaimed water. PS, as the additional carbon source of the ISTY, compared the economic benefits of wastewater treatment with sodium acetate commonly used in wastewater treatment plants. The prices of tap water and reclaimed water refer to the cost set by the Tianjin Water Bureau (0.34 $/m^3^ for reclaimed water, and 0.76 $/m^3^ for domestic water). The price of soluble low molecular organic matter refers to the price of sodium acetate (1003.83 $/t), and the reference price of PS for agricultural waste is 247.10 $/t. According to Section 3.2.1, the dosage of PS is 3.0 g/L, and the dosage of sodium acetate is 0.8 g/L. In Section 2.2.4, the economic benefit analysis shows that, when PS is replaced by traditional sodium acetate as a carbon source, the financial benefit of 0.48 $ can be produced per 1 m^3^ of sewage treatments.

## 4. Conclusions

The work carried out a comprehensive study in agricultural wastes (PS, SD, PT, MT) on the processes of carbon release, denitrification process, and denitrification effect in ISTY. In terms of carbon release, the first-order kinetics was basically followed. PS can stably release a large amount of organic carbon, release TOC within 144 h, accumulate 43.1 ± 0.5 mg/(g·L), and release low nitrogen and phosphorus. Moreover, denitrification could be obviously enhanced by the PS. PS can obviously enhance the nitrogen removal efficiency of the ISTY. After 120 h of operation, the TN removal rate increased from 29.76 to 83.86%, and the accumulation of nitrate nitrogen and nitrite nitrogen was low. Meanwhile, PS supplement increased the abundance ratio of denitrifying bacteria, especially *Pseudomonas* and *Cupriavidus* bacteria, which were related to the denitrification process. The analysis of the economic benefit, of adding a carbon source in the denitrification stage of integrated wastewater treatment, indicates that, when PS is used as a carbon source instead of traditional sodium acetate, the economic benefit of 0.48$ can be generated per 1 m^3^ of wastewater treatments. Therefore, using agricultural waste as a carbon source is the future direction of application.

## Figures and Tables

**Table 1:** Quality and formula of distributed water.

Dispensing Agent	Dosage (g/L)	Trace Element Liquid Composition	Concentration (g/L)	Water Quality Index	Concentration (g/L)
KNO_3_	0.06	FeCl_3_	0.8	NO_3_^−^-N	19.17 ± 0.96
KH_2_PO_4_	0.02	KI	0.18	NH_4_^+^-N	0.54 ± 0.07
MgSO_4_	0.003	CuSO_4_·5H_2_O	0.03	TN	20.14 ± 0.74
NaCl	0.03	ZnCl_2_	0.06	TP	4.73 ± 0.13
trace element	2 mg/L	CoCl_2_·7H_2_O	0.15	COD	11.20 ± 1.21

**Table 2:** Actual sewage quality.

Water Quality Index	TN	NO_3_^−^-N	NH_4_^+^-N	TP	COD
Concentration (g/L)	19.6 ± 0.67	5.9 ± 0.42	9.4 ± 0.36	4.6 ± 0.14	69.6 ± 5.13

**Table 3:** Content of C, N, P, and S elements in different materials.

Material	w%			
	C	N	P	S
PS	44.18 ± 1.22	1.20 ± 0.07	0.18 ± 0.02	0.30 ± 0.05
SD	48.92 ± 0.86	0.22 ± 0.03	0.17 ± 0.04	0.16 ± 0.04
PT	50.04 ± 1.52	2.27 ± 0.08	0.34 ± 0.06	0.39 ± 0.08

PS: peanut shell; SD: sawdust; PT: peat.

**Table 4:** Kinetic fitting of the carbon release process from different carbon sources.

Material	First-Order			Ritger-Peppas		
	Fitting Equation	R^2^	k	Fitting Equation	R^2^	N
PS	$y=217.66\times [1-\exp\left(-0.16x\right)$]	0.99	0.16	$y=107.39\times \left(x^{0.16}\right)$	0.94	0.16
SD	$y=91.16\times [1-\exp\left(-0.25x\right)$]	0.97	0.25	$y=51.89\times \left(x^{0.13}\right)$	0.96	0.13
PT	$y=60.72\times \left[1-\exp\left(-0.16x\right)\right]$	0.91	0.16	$y=27.33\times \left(x^{0.19}\right)$	0.98	0.19
MT	$y=115.08\times \left[1-\exp\left(-0.17x\right)\right]$	0.90	0.17	$y=50.92\times \times \left(x^{0.19}\right)$	0.95	0.19

MT: A mixture of peanut shell, sawdust and peat.

**Table 5:** Nitrogen removal rate equation of PS.

Material	NO_3_^−^-N		TN	
PS	$y=0.99\times \left[1-\exp\left(-0.06x\right)\right]$	R^2^ = 0.99	$y=0.81\times [1-\exp\left(-0.05x\right)$]	R^2^ = 0.97

## Data Availability

Data is contained within the article. For detailed information of each part, please contact the corresponding author.
